# Effect of N-Acetyl-L-Cysteine (NAC) on Inflammation After Intraperitoneal Mesh Placement in an *Escherichia coli* Septic Rat Model: A Randomized Experimental Study

**DOI:** 10.3390/medsci13040318

**Published:** 2025-12-14

**Authors:** Styliani Parpoudi, Ioannis Mantzoros, Orestis Ioannidis, Konstantinos Zapsalis, Thomai Gamali, Dimitrios Kyziridis, Christos Gekas, Elissavet Anestiadou, Savvas Symeonidis, Stefanos Bitsianis, Efstathios Kotidis, Manousos-Georgios Pramateftakis, Dimosthenis Miliaras, Anastasia Bikouli, Georgios Iosifidis, Stamatios Angelopoulos

**Affiliations:** 1 Surgical Breast Oncology Department, Theageneio Hospital, 546 39 Thessaloniki, Greece; stellaparpoudi@hotmail.com; 2 4th Department of Surgery, General Hospital “George Papanikolaou”, Aristotle University of Thessaloniki, 570 10 Exochi, Greece; imanvol@gmail.com (I.M.); thwmh88@gmail.com (T.G.); dkyziridis@gmail.com (D.K.); elissavetxatz@gmail.com (E.A.); simeonidissavvas@yahoo.com (S.S.); sbitsiani@gmail.com (S.B.); skotidis@gmail.com (E.K.); mpramateftakis@hotmail.com (M.-G.P.); saggelopoulos@auth.gr (S.A.); 3 Orthopaedic Department, Ippokrateio Hospital, 546 42 Thessaloniki, Greece; gkekasch@gmail.com; 4 Laboratory of Histology-Embryology, Aristotle University of Thessaloniki, 541 24 Thessaloniki, Greece; miliaras@auth.gr; 5 1st Intensive Care Unit, George Papanikolaou Hospital, 570 10 Thessaloniki, Greece; anastabik@gmail.com; 6 Laboratory of Anatomy, Histology & Embryology, School of Veterinary Medicine, Faculty of Health Sciences, Aristotle University of Thessaloniki, 541 24 Thessaloniki, Greece; iosifidisgeorg@gmail.com

**Keywords:** N-acetyl-L-cysteine (NAC), intraperitoneal mesh, *Escherichia coli* peritonitis, peritoneal adhesions, neutrophil infiltration, fibrosis, neovascularization, cytokines, biofilm modulation, rat experimental model

## Abstract

**Background/Objectives**: The safety of intraperitoneal mesh placement in contaminated fields remains controversial because of the increased risk of inflammation and adhesion formation. N-acetyl-L-cysteine (NAC) has antioxidant, pro-fibrinolytic and antibiofilm actions that could attenuate this response. The aim of this study is to determine whether NAC reduces mesh-related inflammation in a septic model created by intraperitoneal *Escherichia coli* (*E.coli*) inoculation. The primary comparison was prospectively defined between *E. coli*–inoculated animals treated with NAC (D) and those without NAC (B). Groups without *E. coli* (A,C,E) are presented for context and were compared previously. **Methods**: In this randomized, double-blind experimental model (five groups, *n* = 20 per group), all rats underwent midline laparotomy with intraperitoneal placement of a composite mesh, followed by standardized ciprofloxacin administration. The septic groups received intraperitoneal *E. coli*, while the NAC-treated groups additionally received intraperitoneal NAC (150 mg/kg). Serum levels of IL-1α, IL-6, and TNF-α were measured on postoperative days 7, 14, and 21. On day 21, adhesions were graded using the Modified Diamond system, histology (inflammatory infiltration, fibrosis, neovascularization) was scored, and mesh cultures were obtained. Cytokine data were analyzed with repeated-measures ANOVA, while categorical or ordinal outcomes were assessed using χ^2^ or Fisher’s exact tests with Bonferroni-adjusted pairwise comparisons. **Results**: *E. coli* inoculation significantly increased adhesion burden and worsened histologic scores compared with controls (both *p* < 0.001). NAC administration in the septic model significantly reduced adhesions and improved all histologic domains relative to *E. coli* alone (all *p* ≤ 0.003), with values comparable to controls (non-significant across domains). For cytokines, there was a significant overall group effect for IL-1α, IL-6, and TNF-α (all *p* < 0.001), without a main effect of time or time × group interaction. Pairwise contrasts showed lower IL-1α (*p* = 0.024), IL-6 (*p* < 0.001), and TNF-α (*p* < 0.001) levels in group D versus B, and lower IL-6 and TNF-α in group D versus A (both *p* < 0.001). Mesh culture positivity rate was higher in group B than A (*p* < 0.001) and showed a non-significant reduction in group D versus B (*p* = 0.10). No perioperative deaths occurred. **Conclusions**: NAC attenuated septic, mesh-associated inflammation—normalizing adhesions and histology and reducing IL-6 and TNF-α— supporting its role as a host-directed adjunct alongside antibiotics. Further translational studies are warranted to define the optimal dose, timing, and clinical indications.

## 1. Introduction

Mesh reinforcement has become the standard approach in ventral and incisional hernia repair because it reduces recurrence risk compared with primary suture repair; however, its use in contaminated or potentially contaminated operative fields remains controversial due to concerns over infection, fistula formation, and dense adhesions [1,2,3]. Contemporary consensus statements allow synthetic mesh selectively in clean-contaminated settings, yet clinicians remain cautious in the presence of frank sepsis or bacterial inoculation where the peritoneal inflammatory response is amplified and outcomes are unpredictable [1,4].

At a mechanistic level, peritoneal injury triggers mesothelial disruption, fibrin deposition, and a cytokine-driven cascade (notably IL-1α, IL-6, and TNF-α) that promotes leukocyte recruitment, neoangiogenesis, and fibroblast activation, culminating in adhesion formation and foreign-body fibrosis around the mesh [5]. Bacterial contamination exacerbates these processes through pathogen-associated molecular patterns and biofilm formation on prosthetic surfaces, which both sustain inflammation and shield organisms from host defenses and antibiotics [6,7,8]. Thus, strategies that modulate the host inflammatory/fibrinolytic balance and interfere with biofilm architecture may improve outcomes when mesh is required in septic conditions.

N-acetyl-L-cysteine (NAC) is a well-characterized glutathione precursor with antioxidant and anti-inflammatory properties; it can attenuate NF-κB signaling, enhance peritoneal fibrinolysis, and has been shown to impair bacterial biofilms while potentiating antibiotic activity [9,10,11]. At the molecular level, NAC donates a free thiol group that replenishes intracellular glutathione, scavenges reactive oxygen species, and reduces disulfide bonds within extracellular matrices and mucus [12]. Through these redox effects, NAC down-regulates transcription factors such as NF-κB and AP-1, attenuates activation of the NLRP3 inflammasome, and modulates the balance between plasminogen activators and inhibitors, thereby favoring fibrinolysis and limiting persistence of the fibrin scaffold that seeds adhesions. In parallel, NAC can interfere with bacterial biofilm architecture by disrupting extracellular polymeric substance cross-linking, which may weaken biofilm stability and partially restore antibiotic penetration and phagocytic clearance [13]. These mechanisms provide biological plausibility for a protective effect of NAC on mesh-related inflammation in septic conditions.

Preclinical work has reported that NAC reduces postoperative adhesions and mitigates peritoneal inflammatory injury in non-septic models, but data in explicitly contaminated or septic mesh scenarios are limited [9,11]. Given its safety profile and multimodal actions, NAC is an attractive adjunct to standard antibiotic therapy and source control.

The aim of this study is to determine whether NAC attenuates mesh-related inflammation under septic conditions. As an important note, groups not inoculated with *E. coli* are presented to contextualize the model but are not included in the primary analytic contrasts, as this comparison has been previously published [14].

## 2. Materials and Methods

### 2.1. Study Design

Healthy male Wistar rats (10–14 weeks old; ~200–300 g) were used for this study. This was a randomized, prospective, double-blind experimental study in rats, designed to model clean, clean-contaminated, and contaminated (septic) scenarios of intraperitoneal mesh placement. Animals were acclimatized before experimentation and housed in pairs in a controlled facility (18–22 °C; 55–65% humidity; 12 h light/12 h dark cycle) with unrestricted access to standard chow and water, in accordance with EU Directive 86/609, Greek Presidential Decree 160/91, and the ARRIVE recommendations.

### 2.2. Study Groups and Interventions

Animals were allocated in equal numbers (*n* = 20/group) to five prespecified groups, each reflecting a clinically relevant condition and pharmacologic strategy. All animals underwent midline laparotomy and intraperitoneal placement of a composite mesh; group-specific interventions are detailed below.

- Group A (Control, clean field): Mesh placement followed by intraperitoneal ciprofloxacin and 0.9% saline. No deliberate contamination or bowel surgery was performed.
- Group B (Escherichia coli septic field): Mesh placement followed by intraperitoneal inoculation with *E. coli* (200 μL containing 10^6^ CFU) and ciprofloxacin; animals received 0.9% saline as vehicle.
- Group C (Clean-contaminated bowel surgery): Mesh placement plus a standardized wedge colectomy with primary end-to-end anastomosis (6–8 extramucosal 5/0 Prolene^®^ sutures), with ciprofloxacin and 0.9% saline.
- Group D (Septic field + NAC): Identical to Group B (*E. coli* inoculation and ciprofloxacin) with the addition of intraperitoneal N-acetyl-L-cysteine (NAC) at 150 mg/kg (0.2 Ml).
- Group E (Clean-contaminated bowel surgery + NAC): Identical to Group C (wedge colectomy and ciprofloxacin) with the addition of intraperitoneal NAC 150 mg/kg (0.2 mL).

For the present manuscript, the primary analytic contrast isolates the effect of NAC in sepsis (B vs. D). Added prespecified contrasts were B vs. C (sepsis vs. clean-contaminated colectomy without NAC) and D vs. E (sepsis + NAC vs. clean-contaminated + NAC). Groups without *E. coli* inoculation (A, C, E) have been analyzed previously and are included here to contextualize the inflammatory range of the model [14].

### 2.3. Randomization and Masking

One hundred male Wistar rats were randomized in equal blocks to the five groups using a computer-generated sequence. Randomization was implemented using a list with variable block sizes of 5 and 10, prepared by an investigator who was not involved in surgery, postoperative care, or outcome assessment. Allocation codes were placed in sequentially numbered, opaque, sealed envelopes that were opened in the operating room immediately before laparotomy. A priori, animals that died before postoperative day (POD) 21 or lacked evaluable mesh or tissue specimens were to be excluded from the primary analysis but described in a CONSORT-style flow diagram; in practice, no such dropouts occurred. Allocation was concealed from the assessors. Macroscopic adhesion scoring and histopathological grading were performed by observers blinded to group assignment.

### 2.4. Operative and Perioperative Procedures

#### 2.4.1. Anaesthesia and Preparation

General anaesthesia was induced with intraperitoneal ketamine (50 mg/kg) and xylazine (5 mg/kg). The abdominal wall was shaved and prepared with povidone-iodine. A 3 cm midline laparotomy was performed under aseptic conditions with active temperature maintenance.

#### 2.4.2. Mesh Implantation

**Figure 1:**
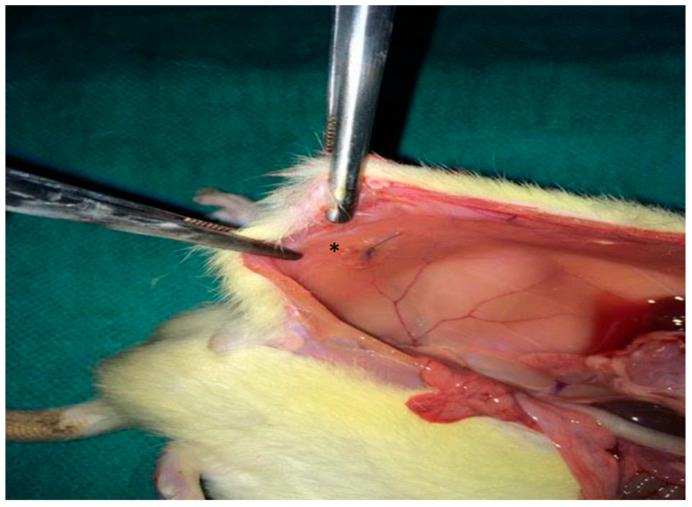
Intraoperative photograph illustrating the placement of the synthetic mesh (*) over the peritoneal defect.

A 2 × 2 cm composite mesh (Ethicon Proceed^®^, polypropylene/polydioxanone/polyglactin, Ethicon LLC, New Jersey, NJ, USA) was placed intraperitoneally on the peritoneal surface of the abdominal wall and fixed with four interrupted 4/0 Prolene^®^ sutures, Ethicon LLC, New Jersey, NJ, USA (Figure 1). The abdominal wall was closed in a single layer with 3/0 silk.

#### 2.4.3. Creation of Contamination Models

In the septic arms (B and D), *E. coli* inoculum (200 μL; 10^6^ CFU) was delivered intraperitoneally after mesh fixation 1. In the clean-contaminated arms (C and E), a wedge colectomy was per-formed approximately 5 cm distal to the caecum, followed by primary end-to-end anastomosis with 6–8 extramucosal 5/0 Prolene^®^ stitches.

### 2.5. Pharmacologic Interventions

All animals received intraperitoneal ciprofloxacin (1 mL of a 2 mg/mL solution) as standardized antibiotic coverage. NAC (150 mg/kg in 0.2 mL) was administered intraperitoneally in the NAC-designated groups (D and Ε), based on the protocol established in the underlying doctoral work and prior literature [9,10,11]. The chosen NAC dose and single intraperitoneal administration were informed by previous rat studies of intra-abdominal inflammation and adhesion formation in which doses between 100 and 300 mg/kg achieved robust antioxidant and pro-fibrinolytic effects without compromising anastomotic healing or survival [15]. The current regimen therefore represents a mid-range, biologically active dose with an acceptable safety margin for this model.

### 2.6. Postoperative Care

Animals recovered in clean cages with ad libitum access to food and water and were monitored at least once daily. Analgesia and supportive measures followed institutional practice. Any animal exhibiting distress was evaluated and treated by veterinary staff.

### 2.7. Euthanasia

On postoperative day (POD) 21, animals were euthanized under deep anaesthesia by intracardiac potassium chloride injection, according to predefined humane endpoints.

### 2.8. Outcome Measures

Primary endpoints for this manuscript were (i) systemic inflammatory cytokines (IL-1α, IL-6, TNF-α) measured on POD 7, 14, and 21; and (ii) macroscopic adhesion burden and histological injury at POD 21. Secondary endpoints included mesh-associated microbiology at explant.

#### 2.8.1. Cytokine Assays

Survival blood samples were obtained under light anaesthesia on POD 7 and 14 via peripheral venipuncture (tail/lateral vein), with terminal sampling on POD 21 performed immediately prior to euthanasia by cardiac puncture under deep anaesthesia. Whole blood was allowed to clot at room temperature, then centrifuged; serum was aliquoted in duplicate and stored at −80 °C until batch analysis. Assays were run in duplicate; samples outside the dynamic range were diluted and re-assayed; standard curves were fit with a 4-parameter logistic model. Serum IL-1α, IL-6, and TNF-α were quantified using validated ELISA kits (Sigma-Aldrich; RAB0272, RAB0311, RAB0479) per manufacturer protocol (intra-assay CV < 10%, inter-assay CV < 12%; detection limits ~15 pg/mL, 30 pg/mL, and 25 pg/mL, respectively).

#### 2.8.2. Macroscopic Adhesions

At POD 21, the abdomen was reopened and adhesions were assessed by a blinded assessor using the Modified Diamond scale [16], recording extent, tenacity, and tissue involvement over the mesh surface. The scale was applied as follows: grade 0—no adhesions; grade 1—thin, filmy adhesions easily lysed by blunt traction; grade 2—firm adhesions requiring careful blunt/sharp dissection, limited in area; grade 3—dense, vascularized adhesions with broad attachment and/or visceral involvement requiring sharp dissection. The anatomic location and the percentage of mesh surface involved were documented on a standardized case report form, with representative photographs.

#### 2.8.3. Histopathology

The mesh–abdominal wall complex was excised en bloc, oriented, and immersed in 10% neutral-buffered formalin for fixation prior to routine paraffin processing. Sections (~2–3 µm) were stained with haematoxylin–eosin (H&E). Blinded observers applied a 0–3 semiquantitative scale for: inflammatory cell (neutrophil-predominant) infiltration, fibrosis (collagen deposition/organization and thickness of fibrous layer), and neo-vascularization (density and maturity of microvessels traversing the neoperitoneum overlying the mesh). Scores were assigned using predefined morphological criteria from the thesis protocol, with adjudication in case of discrepancy. To ensure reproducibility of histological assessments, two independent blinded observers (experienced histopathologists) evaluated all slides using the predefined 0–3 semiquantitative scales for inflammatory infiltration, fibrosis, and neovascularization. Each slide was scored separately by both observers, and discrepancies of more than one grade were resolved through joint review and consensus. Interobserver agreement for the three domains was assessed using weighted Cohen’s kappa, which demonstrated substantial agreement across parameters (κ = 0.72 for inflammation, κ = 0.76 for fibrosis, κ = 0.70 for neovascularization), indicating high reproducibility of the scoring methodology. All analyses were performed with observers fully blinded to group allocation to minimize assessment bias.

#### 2.8.4. Microbiology

The mesh–abdominal wall complex was excised en bloc, oriented, and immersed in 10% neutral-buffered formalin for fixation prior to routine paraffin processing. Sections (~2–3 µm) were stained with haematoxylin–eosin (H&E). Blinded observers applied a 0–3 semiquantitative scale for: inflammatory cell (neutrophil-predominant) infiltration, fibrosis (collagen deposition/organization and thickness of fibrous layer), and neo-vascularization (density and maturity of microvessels traversing the neoperitoneum overlying the mesh). Scores were assigned using predefined morphological criteria from the thesis protocol, with adjudication in case of discrepancy.

After macroscopic assessment, the mesh–abdominal wall complex was explanted under aseptic conditions. Using sterile instruments, the mesh surface and adjacent tissue were gently rinsed with sterile saline and then vortexed in 5 mL of broth; aliquots were plated on blood and MacConkey agar and incubated aerobically at 37 °C for 24–48 h. Growth of *E. coli* or other organisms was recorded as positive or negative for each animal. For the purposes of this analysis, culture results were dichotomized (positive/negative) and compared between groups, rather than analyzed semi-quantitatively, because the primary interest was the presence or absence of persistent contamination.

### 2.9. Sample Size and Statistical Analysis

Categorical variables (adhesion and histologic grades, culture positivity) were compared using χ^2^ or Fisher’s exact tests. Continuous variables (cytokines) were evaluated for distribution (Kolmogorov–Smirnov; Shapiro–Wilk) and analyzed with repeated-measures ANOVA, including Mauchly’s test of sphericity; when violated, Greenhouse–Geisser corrections were applied. Planned pairwise contrasts used Bonferroni adjustment. Two-sided *p* < 0.05 denoted statistical significance. Analyses were performed in SPSS v23 and R v4.0.3. The total sample (*n* = 100; 20/group) derived from the doctoral protocol and provided adequate precision for detecting group differences in the primary inflammatory endpoints while adhering to ethical use of animals. Continuous cytokine measurements are summarized as means ± standard deviation and 95% confidence intervals in the text and accompanying tables to facilitate critical appraisal of group differences.

### 2.10. Animal Housing and Welfare

All animals were healthy male Wistar rats (10–14 weeks old; 200–300 g) acclimatized for at least one week before experimentation. They were housed in pairs in individually ventilated cages under controlled environmental conditions (temperature 18–22 °C, relative humidity 55–65%, 12 h light/12 h dark cycle), with free access to standard laboratory chow and water. Bedding was changed regularly, and environmental enrichment was provided according to institutional practice. Animals were monitored at least once daily for general condition, food and water intake, wound status, and signs of discomfort or distress. Any animal exhibiting pain or abnormal behavior was evaluated and treated by veterinary staff in accordance with predefined humane endpoints.

### 2.11. Ethical Approval

All procedures complied with EU Directive 86/609 and Greek Presidential Decree 160/91 and were approved by the competent Veterinary Service (approval No. 23962/121). The study was designed and reported in accordance with the ARRIVE guidelines to ensure comprehensive and transparent reporting of animal research [17]. Key methodological items recommended by ARRIVE (study design, randomization, blinding, sample size determination, and statistical methods) are explicitly described in the sections above to facilitate reproducibility and ethical evaluation.

## 3. Results

Detailed numerical distributions for all macroscopic, histological, and cytokine outcomes are provided in Supplementary Tables S1–S7.

### 3.1. Cohort and Protocol Adherence

All randomized animals underwent the assigned procedures without intra-operative mortality; complete 21-day follow-up was achieved with no protocol deviations that affected primary endpoints. Perioperative recovery, weight, and behavior remained within expected ranges for this model. No animals were excluded from the primary analyses for missing data, mortality, or technical failures.

### 3.2. Adhesions

A significant overall difference in adhesion burden was observed among groups (omnibus *p* < 0.001). Compared with controls (Group A), *E. coli* inoculation (Group B) resulted in a marked increase in adhesion severity (A vs. B: *p* < 0.001). The addition of NAC in the septic model (Group D) significantly reduced adhesions compared with Group B (B vs. D: *p* < 0.001) and showed no difference from controls (A vs. D: non-significant).

Group B also exhibited more severe adhesions than the clean-contaminated colectomy without NAC (Group C; B vs. C: *p* < 0.05), indicating a progressive gradient from clean-contaminated to septic conditions. When NAC was administered, adhesion formation under both septic (Group D) and clean-contaminated (Group E) settings became comparable (D vs. E: non-significant), with both approximating control levels.

**Figure 2:**
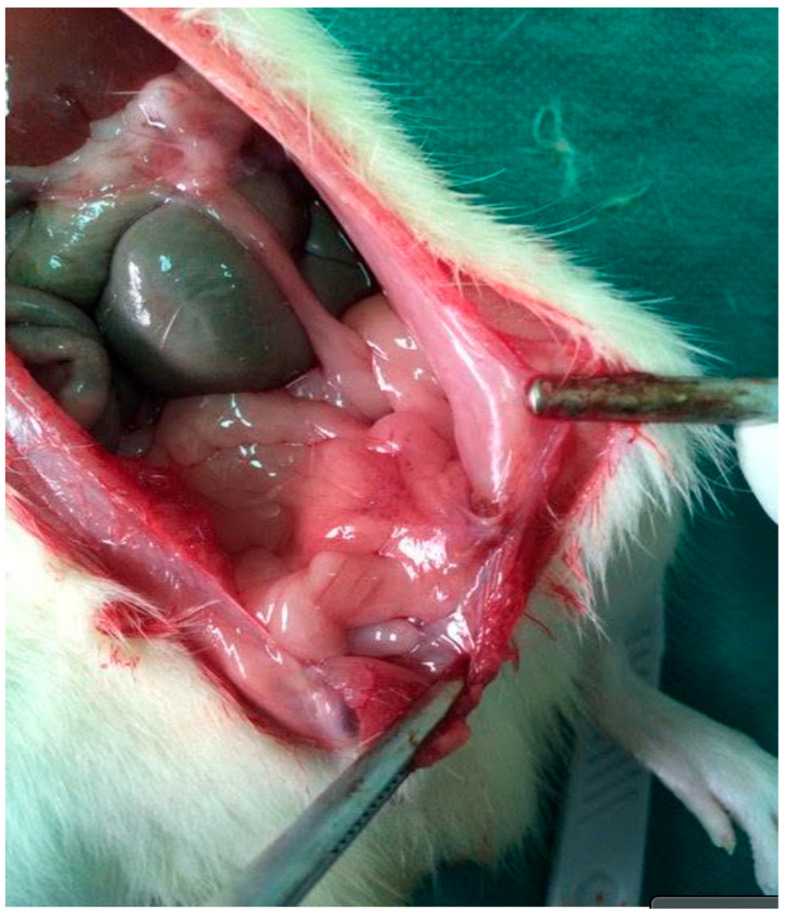
Re-entry view demonstrating extensive intra-abdominal adhesions on postoperative day 21. Dense fibrous bands are visible between the parietal peritoneum and adjacent intestinal loops, consistent with high-grade adhesion formation in the septic group.

**Figure 3:**
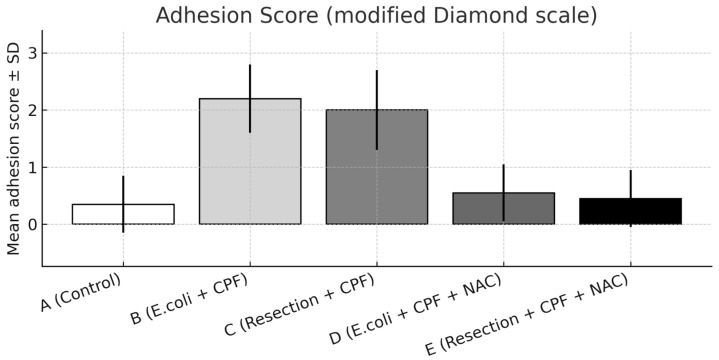
Adhesion severity on postoperative day 21 scored using the modified Diamond scale, presented as mean ± SD across experimental groups. Statistical significance: A vs. B *p* < 0.001; B vs. D *p* < 0.001; B vs. C *p* < 0.05; A vs. D and D vs. E, not significant.

Distributionally, grade 2–3 adhesions were absent in Group D and uncommon in Groups A and E, whereas Group B concentrated the highest grades, reflecting the marked reduction in adhesion severity achieved with NAC. Representative images are provided in Figure 2 (extensive adhesions on day 21) and Figure 3 (distributional shift toward low-grade adhesions with NAC).

### 3.3. Histology

Blinded semiquantitative scoring revealed significant between-group differences across all evaluated domains—inflammatory cell infiltration, fibrosis, and neovascularization (all omnibus *p* < 0.01). Pairwise comparisons mirrored the macroscopic adhesion patterns:

- Neutrophil infiltration: B > A (*p* < 0.001); D < B (*p* < 0.001); A vs. D non-significant. Additional contrasts: B > C (*p* < 0.05); D vs. E non-significant.
- Fibrosis: A vs. B (*p* = 0.002); B vs. D (*p* = 0.002); A vs. D non-significant; B vs. C (*p* < 0.05); D vs. E non-significant.
- Neovascularization: A vs. B (*p* < 0.001); B vs. D (*p* = 0.003); A vs. D non-significant; B vs. C (*p* < 0.05); D vs. E non-significant.

**Figure 4:**
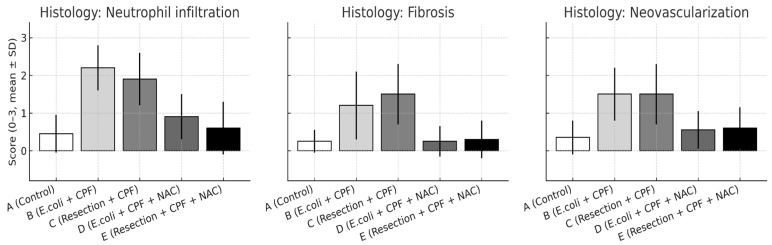
Histological scores for neutrophil infiltration, fibrosis, and neovascularization on postoperative day 21 are shown as mean ± SD across experimental groups. Statistical significance: A vs. B *p* < 0.001; B vs. D *p* ≤ 0.003; A vs. D and D vs. E, not significant; B vs. C *p* < 0.05.

Qualitatively, septic animals (Group B) demonstrated dense, neutrophil-rich infiltrates, thick collagenous encapsulation, and abundant microvascular proliferation within the neoperitoneum overlying the mesh. In contrast, NAC treatment (Group D) normalized these changes, resembling the fine, pauci-cellular fibrous layer and sparse microvessels characteristic of controls (Group A). Groups C and E exhibited intermediate, low-grade injury patterns consistent with clean-contaminated manipulation rather than frank peritonitis (Figure 4).

### 3.4. Serum Cytokines

Blinded semiquantitative scoring demonstrated significant between-group differences across all evaluated tissue domains—inflammatory cell infiltration, fibrosis, and neovascularization (all omnibus *p* < 0.01). Pairwise comparisons mirrored the macroscopic findings:

- Neutrophil infiltration: B > A (*p* < 0.001); D < B (*p* < 0.001); A vs. D non-significant. Additional contrasts: B > C (*p* < 0.05); D vs. E non-significant.
- Fibrosis: A vs. B (*p* = 0.002); B vs. D (*p* = 0.002); A vs. D non-significant; B vs. C (*p* < 0.05); D vs. E non-significant.
- Neovascularization: A vs. B (*p* < 0.001); B vs. D (*p* = 0.003); A vs. D non-significant; B vs. C (*p* < 0.05); D vs. E non-significant.

There was a significant overall group effect for all three cytokines across the 21-day period (IL-1α, IL-6, TNF-α; omnibus *p* < 0.001 for each), with no main effect of time and no time × group interaction, indicating relatively stable group trajectories once animals were allocated to their respective conditions. In pairwise contrasts, septic animals without NAC (Group B) exhibited higher IL-1α, IL-6, and TNF-α concentrations than clean controls (Group A; all *p* < 0.01). Adjunctive NAC in the septic model (Group D) significantly reduced IL-1α (D vs. B: *p* = 0.024), IL-6 (*p* < 0.001), and TNF-α (*p* < 0.001) relative to Group B, with levels that were not different from controls (A vs. D: non-significant). Compared with clean-contaminated surgery without NAC (Group C), Group B again showed higher IL-6 and TNF-α (*p* < 0.05), whereas the two NAC-treated groups (D and E) were similar to each other and closely approximated the low-inflammation profile of Group A. Overall, NAC primarily shifted the systemic inflammatory profile downward in septic animals rather than altering the temporal pattern of cytokine release.

To explore whether distinct responder subgroups existed within treatment arms, we assessed the distribution of IL-1α, IL-6, and TNF-α values at each time point. Cytokine levels demonstrated moderate intra-group variability consistent with expected biological dispersion in this model; however, no bimodal patterns, outliers suggestive of divergent responders, or subgroup-like clusters were observed. Variability remained narrower in the NAC-treated septic group (D) than in untreated sepsis (B), reflecting the overall dampened inflammatory trajectory induced by NAC.

**Figure 5:**
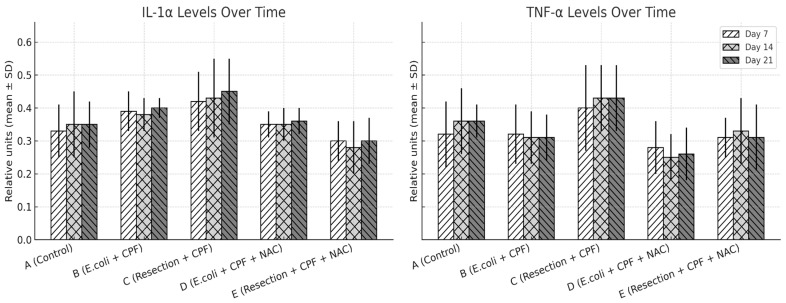
IL-1α and TNF-α expression levels (mean ± SD) on POD 7, 14, and 21 across treatment groups, quantified from peritoneal fluid samples using ELISA, with grayscale patterns representing time points. Statistical significance: A vs. B *p* ≤ 0.002; B vs. D *p* ≤ 0.003; A vs. D and D vs. E, not significant; B vs. C *p* < 0.05.

Qualitatively, septic animals (Group B) showed dense, neutrophil-rich infiltrates, thick collagenous encapsulation, and abundant microvascular proliferation within the neoperitoneum overlying the mesh. In contrast, NAC treatment (Group D) normalized these alterations, approximating the fine, pauci-cellular fibrous layer and sparse microvasculature typical of controls (Group A). Groups C and E exhibited intermediate, low-grade injury patterns consistent with clean-contaminated manipulation rather than frank peritonitis (Figure 5).

**Figure 6:**
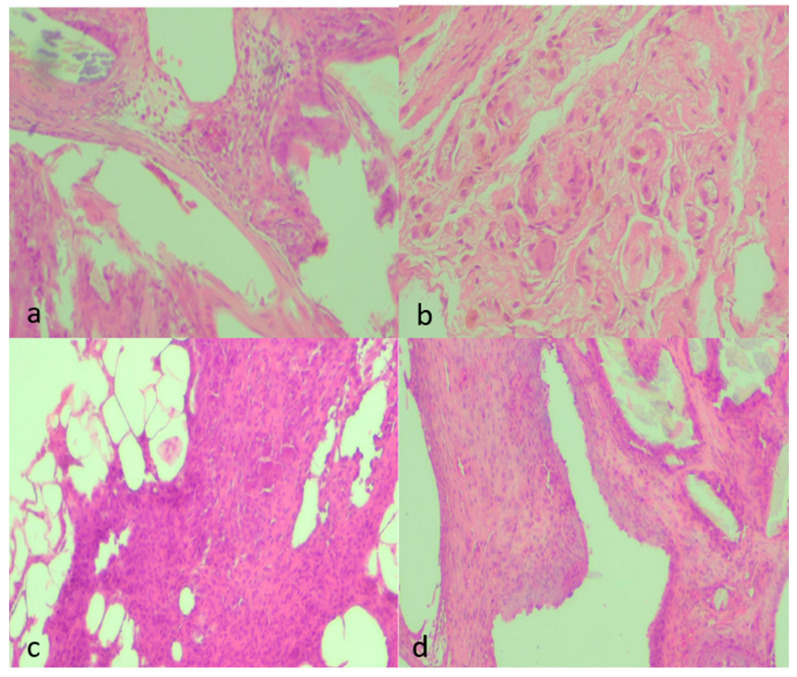
Representative histological micrographs showing fibrosis, inflammation, and neovascularization across experimental groups (H&E, ×100). (**a**) Moderate fibrosis (score 2) characterized by moderately thick collagen bundles, moderate inflammatory infiltrate (2), and mild neovascularization (1) with few newly formed capillaries. (**b**) Marked fibrosis (3) with dense, compact collagen deposition, sparse inflammatory cells (1), and minimal neovascularization (1). (**c**) Mild fibrosis (1) with thin collagen strands, moderate inflammatory infiltrate (2) consisting mainly of mononuclear cells, and moderate neovascularization (2) with visible small-caliber neovessels. (**d**) Moderate fibrosis (2) with interlacing collagen, moderate inflammatory infiltrate (2), and moderate neovascularization (2) with loosely distributed microvessels. All images were captured under identical conditions (H&E, ×100), ensuring consistent spatial representation across panels.

Qualitative findings were further supported by representative histological micrographs illustrating the spectrum of tissue responses across groups (Figure 6a–d). Sections from septic animals demonstrated dense inflammatory infiltrates and moderate fibrosis, whereas clean-contaminated conditions showed milder collagen deposition with limited vascular proliferation. NAC administration notably attenuated these changes, with specimens from the treated septic and clean-contaminated groups displaying thin, well-organized fibrous layers and sparse neovascularization, consistent with the semiquantitative scores.

### 3.5. Microbiology and Follow-Up

Mesh cultures were more frequently positive in B than A (A vs. B: *p* < 0.001). NAC showed a non-significant reduction in culture positivity (B vs. D: *p* = 0.10; A vs. D: *p* = 0.09), consistent with a host-directed, non-bactericidal mechanism. B vs. C favored C (*p* < 0.05), and D vs. E was non-significant, mirroring the tissue and cytokine readouts. Collectively, microbiology did not alter the principal inference that NAC primarily modulates inflammation rather than sterilizes the field. This pattern suggests that ciprofloxacin provided the main bactericidal effect, whereas NAC may have contributed indirectly through biofilm disruption and modulation of host defenses without fully eliminating viable bacteria from the mesh surface.

## 4. Discussion

This study delineates a graded inflammatory response to intraperitoneal mesh across clean, clean-contaminated, and septic conditions, and shows that N-acetyl-L-cysteine (NAC) selectively mitigates the septic phenotype. Escherichia coli inoculation (B) amplified peritoneal inflammation at macroscopic, microscopic, and systemic levels, whereas adjunctive NAC (D) normalized these readouts toward the clean control (A). Adhesion burden, neutrophil-rich infiltration, fibrosis, and neovascularization were all heightened with sepsis and were consistently attenuated by NAC, while circulating IL-6 and TNF-α fell below control levels under NAC exposure. Culture positivity, however, declined only numerically with NAC and did not reach statistical significance, underscoring a host-directed rather than bactericidal principal mechanism. From a mechanistic perspective, these findings are compatible with the known redox-modulating, pro-fibrinolytic, and antibiofilm properties of NAC described in the introduction.

Positioning the septic signal in contrast to clean-contaminated bowel surgery highlights the clinical severity gradient represented in this experimental model. Sepsis without NAC (B) exceeded clean-contaminated colectomy without NAC (C) in adhesion severity, histologic injury, and cytokine levels, indicating that overt contamination—not bowel handling per se—is the dominant driver of adhesiogenesis and tissue damage. Under NAC exposure, septic and clean-contaminated conditions converged: D and E were statistically indistinguishable across tissue domains and cytokines, both approximating the low-inflammation profile of group A. Taken together, these comparisons support the interpretation that NAC restores fibrinolytic balance and blunts redox-driven and cytokine signaling in sepsis, thereby collapsing the inflammatory gap between frank contamination and clean-contaminated injury [18].

These results are biologically plausible within the well-characterized cascade of peritoneal injury and adhesiogenesis. Following mesothelial disruption and foreign-body exposure, an early fibrinous matrix is laid down while peritoneal fibrinolysis is transiently suppressed; concomitant cytokine surges (IL-1α, IL-6, TNF-α) recruit neutrophils and monocyte-derived macrophages, drive angiogenesis, and activate fibroblasts, thereby promoting maturation of adhesions and encapsulating fibrosis around the prosthesis [5]. Bacterial contamination augments this process via pathogen-associated molecular patterns and rapid biofilm formation on polymer surfaces, which sustains inflammatory signaling and impedes antibiotic penetration [8,19,20]. Against this pathophysiological backdrop, the pattern observed with NAC—normalization of histology and macroscopic adhesions despite unchanged inoculation status—supports a mechanism that restores fibrinolytic balance and dampens redox-driven inflammatory amplification rather than direct bactericidal activity.

Several preclinical studies have examined NAC or alternative host-directed agents in models of peritonitis, sepsis, or abdominal surgery [14]. In non-septic adhesion models, NAC, statins, and antioxidant vitamins have each reduced adhesion scores to varying degrees [21], whereas physical barrier agents such as hyaluronic-acid/carboxymethylcellulose membranes or icodextrin solutions act mainly by mechanically separating injured surfaces [22].Compared with these reports, the present work is distinctive in combining a septic mesh model with systemic cytokine profiling and microbiological readouts, and the magnitude of benefit observed with NAC on both adhesions and histology appears broadly comparable to, or greater than, the effects described for other pharmacologic modulators in non-septic settings.

The mechanistic rationale for NAC in this setting is multifaceted and is well described. As a glutathione precursor, NAC replenishes intracellular antioxidant capacity and blunts reactive oxygen species–mediated activation of pro-inflammatory pathways such as NF-κB, with downstream reductions in IL-6 and TNF-α [23,24,25,26]. In parallel, NAC has been shown to tilt the peritoneal environment toward fibrinolysis—through effects on the plasminogen activator system and suppression of antifibrinolytic mediators—thereby limiting persistence of the initial fibrin scaffold that seeds adhesions [27]. A complementary line of evidence indicates that NAC can interfere with bacterial biofilm architecture and extracellular polymeric substance assembly, thereby reducing biofilm stability and restoring some antibiotic susceptibility. The present findings—improvements in cytokines and tissue endpoints with only a non-significant trend in culture positivity—fit the profile of a host-directed adjunct that complements, rather than replaces, antibiotic therapy. Importantly, all animals in this study received ciprofloxacin, and the combination of a strong anti-inflammatory signal with only a modest, non-significant reduction in culture positivity is compatible with pharmacologic synergy (through biofilm disruption and improved antibiotic penetration) rather than antagonism. NAC should therefore be viewed as one component of a multimodal bundle—source control plus antibiotics plus host-directed modulation—rather than as a stand-alone antimicrobial strategy.

The cytokine time-course further supports a treatment-driven rather than time-driven effect. Repeated-measures analyses demonstrated a strong group effect across IL-1α, IL-6 and TNF-α without a main effect of time or time × group interaction, suggesting that allocation to septic versus septic-plus-NAC arms set a relatively stable inflammatory trajectory over the 21-day period. The concordant normalization of neutrophil infiltration, fibrosis and neovascularization mirrors this systemic signal at the tissue level and is consistent with the linked biology of oxidative stress, fibrinolysis, and matrix remodeling [28]. Although the present work was not powered for detailed subgroup analyses, inspection of individual cytokine trajectories suggested relatively homogeneous responses within groups, with no obvious bimodal “responder/non-responder” pattern; future, larger studies could formally explore patient-level predictors of NAC benefit.

These data should be interpreted within the broader clinical debate on mesh use in contaminated and potentially contaminated fields. Contemporary guidance allows synthetic mesh selectively in clean-contaminated settings, yet surgeons remain cautious in septic environments because of infection risk, fistulization, and dense adhesions [29,30,31,32]. The present model shows that, in the context of standardized antibiotic coverage and source control procedures, adjunctive NAC can shift a septic inflammatory profile toward that of uninfected controls across macroscopic, microscopic, and systemic endpoints. While culture positivity did not change significantly, the magnitude and consistency of the anti-inflammatory signal argue that pharmacologic host modulation could expand the safety margin for intraperitoneal reinforcement when contamination cannot be fully avoided [33,34].

Operative and material considerations likely modulate the magnitude of benefit. Meshes differ in polymer composition, pore architecture, coatings, and degradation profiles, and these features shape peritoneal responses—especially under peritonitis conditions [35,36,37,38]. The composite intraperitoneal mesh used here offers a relevant benchmark, but generalization across mesh classes requires direct comparisons in contaminated models. Similarly, prior anti-adhesion agents (barriers, solutions) have shown variable efficacy in peritonitis [39]; situating NAC along-side these strategies highlights that multi-target approaches (oxidative stress, fibrinolysis, neoangiogenesis, and biofilm) may be necessary to deliver robust protection [40,41]. The thesis also underscores surgical technique fac-tors—atraumatic tissue handling, meticulous hemostasis, and peritoneal moisture preservation—that might synergize with NAC to minimize adhesiogenesis [42].

From a translational standpoint, NAC has extensive clinical experience and a favorable safety/PK profile via oral and intravenous routes [43,44]. Bridging work is needed to define perioperative dosing that achieves effective peritoneal concentrations without impairing normal healing, particularly in the presence of anastomoses. The timing of administration is likely important: evidence summarized in the thesis suggests that early intervention during the acute inflammatory window maximizes effects on fibrinolysis and oxidative stress [45,46]. Because all animals received ciprofloxacin, future studies should also map potential pharmacodynamic interactions—beneficial or adverse—between NAC and multidrug regimens used for intra-abdominal sepsis [44,47]. Beyond single-agent therapy, combination strategies pairing NAC with physical anti-adhesion barriers or agents targeting angiogenesis and fibroplasia may offer additive or synergistic benefits [48,49].

## 5. Strengths, Limitations and Future Perspectives

Strengths of this study include randomized allocation, blinded outcome assessment, validated and clinically meaningful endpoints (Modified Diamond adhesions; semiquantitative histology), repeated-measures cytokine profiling with low assay variability, and complete follow-up without perioperative mortality. Limitations include the use of a single species and sex, one mesh type, a single NAC dose and timing schedule, a single organism inoculum, and a 21-day horizon; histology was semiquantitative. Universal ciprofloxacin administration reflects clinical practice but may have attenuated microbiological contrasts, and the study was powered for inflammatory endpoints rather than bacteriologic clearance [19]. Moreover, the sample size was not designed for formal subgroup or individual-variability analyses, so potential heterogeneity of NAC effect across different host phenotypes could not be robustly explored. These considerations reinforce that the principal signal here is anti-inflammatory/anti-adhesive rather than antimicrobial and that the conclusions should be extrapolated cautiously beyond the specific conditions tested. Finally, although extended molecular analyses and additional biomarkers would have strengthened the mechanistic interpretation, these assays were not performed due to economic constraints of the project. We acknowledge this as a limitation, and such molecular profiling represents a clear future direction to complement the present findings.

Future work should (i) define NAC dose–response and timing windows; (ii) test performance across polymer classes and coatings in septic and polymicrobial models; (iii) explore combination regimens with anti-adhesion barriers and agents that modulate fibrinolysis, angiogenesis, or matrix turnover; (iv) perform pharmacokinetic/pharmacodynamic (PK/PD) studies (pharmacokinetic, i.e., how the drug is absorbed, distributed, metabolized, and eliminated; pharmacodynamic, i.e., how drug concentration relates to biological effect) to inform perioperative routes and schedules; and (v) design pragmatic clinical trials where NAC is incorporated into standardized care bundles (source control + antibiotics + host-directed modulation), with endpoints that include adhesions, bowel obstruction, re-operation, surgical-site infection, pain, and quality of life. Additional work incorporating molecular biomarkers of oxidative stress, fibrinolysis, and biofilm disruption, ideally reported in supplementary datasets, would further clarify mechanisms of action. Finally, larger preclinical and early-phase clinical studies could evaluate whether specific subgroups—such as animals or patients with heightened baseline inflammatory or pro-fibrotic profiles—derive disproportionate benefit from NAC-based strategies.

## 6. Conclusions

In this controlled intraperitoneal mesh model, Escherichia coli inoculation produced a robust inflammatory phenotype characterized by increased adhesion burden, intensified neutrophil infiltration, greater fibrosis, and heightened neovascularization. Adjunctive N-acetyl-L-cysteine (group D) consistently mitigated these effects: adhesions and histologic injury returned to levels comparable with uninfected controls, and systemic inflammatory signaling (IL-6 and TNF-α) was reduced relative to untreated septic animals. Mesh culture positivity showed a non-significant decline with NAC, indicating that the principal benefit is host-directed modulation rather than bactericidal activity and that NAC should be considered an adjunct—not a substitute—for antibiotics and source control. Groups without *E. coli* (A, C, Ε) were compared in prior work and are referenced here to contextualize the range of inflammatory responses; the primary inference of this manuscript rests on the septic comparison between groups B and D.

Taken together, these findings support NAC as a promising perioperative adjunct to attenuate mesh-associated inflammation in contaminated settings. Translation will require dose-finding and timing studies, evaluation across mesh types and polymicrobial models, and early-phase clinical trials embedded within standardized care pathways to determine efficacy, safety, and indications. Clarifying the interaction between NAC and standard antibiotic regimens and identifying which patients are most likely to benefit will be key steps toward rational and safe clinical implementation.

## Data Availability

The original contributions presented in this study are included in the Supplementary Materials. Further inquiries can be directed to the corresponding author.

## Supplementary Materials

The following supporting information can be downloaded at: https://www.mdpi.com/article/10.3390/medsci13040318/s1. Table S1. Adhesion Scores Across Experimental Groups. Table S2. Neutrophil Infiltration Scores Across Experimental Groups. Table S3. Fibrosis Scores Across Experimental Groups. Table S4. Neovascularization Scores Across Experimental Groups. Table S5. IL-1α Concentrations (Mean ± SD) at T7, T14, and T21. Table S6. IL-6 Concentrations (Mean ± SD) at T7, T14, and T21. Table S7. TNF-α Concentrations (Mean ± SD) at T7, T14, and T21.

## Abbreviations

The following abbreviations are used in this manuscript:

NAC	N-acetyl-L-cysteine
*E. coli*	*Escherichia coli*
IL-1α	Interleukin-1 alpha
IL-6	Interleukin-6
TNF-α	Tumor necrosis factor alpha
NF-κB	Nuclear factor kappa B
CFU	Colony-forming units
POD	Postoperative day
H&E	Hematoxylin and eosin
ELISA	Enzyme-linked immunosorbent assay
ANOVA	Analysis of variance
SPSS	Statistical Package for the Social Sciences
ARRIVE	Animal Research: Reporting of In Vivo Experiments
EU	European Union
PK	Pharmacokinetics
PD	Pharmacodynamics
CV	Coefficient of variation
R	R Statistical Software
WSES	World Society of Emergency Surgery
