# Supplementary material for: Effect of N-Acetyl-L-Cysteine (NAC) on Inflammation After Intraperitoneal Mesh Placement in an Escherichia coli Septic Rat Model: A Randomized Experimental Study
Source: Med Sci (Basel). 2025 Dec 14;13(4):318. doi: 10.3390/medsci13040318 (PMC12734425; doi:10.3390/medsci13040318)
Supplement: Supplementary file 1 [file medsci-13-00318-s001.zip › medsci-3991313-supplementary.pdf]

Supplementary Material

These tables present detailed statistical summaries of macroscopic, histological, and cytokine outcomes for all experimental groups. Intended as Supplementary Tables S1–S7.

Supplementary Table S1. Adhesion Scores Across Groups

Adhesion Score	Group A	Group B	Group C	Group D	Group E	p-value
0	13 (65%)	0	0	9 (45%)	11 (55%)	<0.001
1	7 (35%)	2 (10%)	6 (30%)	11 (55%)	9 (45%)	0.02–0.52
2	0	12 (60%)	8 (40%)	0	0	<0.001
3	0	6 (30%)	6 (30%)	0	0	0.52; <0.001

Supplementary Table S2. Neutrophil Infiltration Scores

Score	Group A	Group B	Group C	Group D	Group E	p-value
0	11 (55%)	0	0	5 (25%)	10 (50%)	<0.001
1	9 (45%)	2 (10%)	7 (35%)	12 (60%)	8 (40%)	0.058–0.349
2	0	13 (65%)	8 (40%)	3 (15%)	2 (10%)	0.138–<0.001
3	0	5 (25%)	5 (25%)	0	0	0.264–<0.001

Supplementary Table S3. Fibrosis Scores

Score	Group A	Group B	Group C	Group D	Group E	p-value
0	16 (80%)	5 (25%)	2 (10%)	16 (80%)	15 (75%)	0.002

Supplementary Table S4. Neovascularization Scores

Score	Group A	Group B	Group C	Group D	Group E	p-value
0	14 (70%)	2 (10%)	2 (10%)	11 (55%)	11 (55%)	<0.001
1	6 (30%)	6 (30%)	8 (40%)	8 (40%)	8 (40%)	<0.001
2	0	7 (35%)	8 (40%)	1 (5%)	1 (5%)	0.43–<0.001
3	0	1 (5%)	2 (10%)	0	0	0.003

Supplementary Table S5. IL-1α Levels (Mean ± SD) at T7, T14, T21

Group	T7	T14	T21
A	0.33±0.08	0.35±0.10	0.35±0.07
B	0.39±0.06	0.38±0.05	0.44±0.03
C	0.42±0.09	0.43±0.12	0.45±0.10
D	0.35±0.04	0.35±0.05	0.36±0.04
E	0.30±0.06	0.28±0.07	0.30±0.07

Supplementary Table S6. IL-6 Levels (Mean ± SD) at T7, T14, T21

Group	T7	T14	T21
A	0.33±0.08	0.34±0.10	0.38±0.09
B	0.40±0.07	0.37±0.07	0.38±0.08
C	0.43±0.10	0.43±0.12	0.46±0.11
D	0.27±0.05	0.25±0.07	0.28±0.07
E	0.30±0.07	0.29±0.09	0.34±0.07

Supplementary Table S7. TNF-α Levels (Mean ± SD) at T7, T14, T21

Group	T7	T14	T21
A	0.32±0.01	0.36±0.10	0.36±0.05
B	0.32±0.09	0.31±0.08	0.31±0.07
C	0.37±0.11	0.38±0.10	0.40±0.09
D	0.28±0.08	0.25±0.07	0.26±0.06
E	0.31±0.06	0.33±0.07	0.33±0.07
